# RNA-Seq of Kaposi’s sarcoma reveals alterations in glucose and lipid metabolism

**DOI:** 10.1371/journal.ppat.1006844

**Published:** 2018-01-19

**Authors:** For Yue Tso, Andrew V. Kossenkov, Salum J. Lidenge, Owen Ngalamika, John R. Ngowi, Julius Mwaiselage, Jayamanna Wickramasinghe, Eun Hee Kwon, John T. West, Paul M. Lieberman, Charles Wood

**Affiliations:** 1 Nebraska Center for Virology and the School of Biological Sciences, University of Nebraska-Lincoln, Lincoln, Nebraska, United States of America; 2 Wistar Institute, Philadelphia, Pennsylvania, United States of America; 3 Ocean Road Cancer Institute, Dar es Salaam, Tanzania; 4 Muhimbili University of Health and Allied Sciences, Dar es Salaam, Tanzania; 5 Dermatology and Venereology section, University Teaching Hospitals, University of Zambia School of Medicine, Lusaka, Zambia; University of Utah, UNITED STATES

## Abstract

Kaposi’s sarcoma-associated herpesvirus (KSHV) is the etiologic agent of Kaposi’s sarcoma (KS). It is endemic in a number of sub-Saharan African countries with infection rate of >50%. The high prevalence of HIV-1 coupled with late presentation of advanced cancer staging make KS the leading cancer in the region with poor prognosis and high mortality. Disease markers and cellular functions associated with KS tumorigenesis remain ill-defined. Several studies have attempted to investigate changes of the gene profile with in vitro infection of monoculture models, which are not likely to reflect the cellular complexity of the in vivo lesion environment. Our approach is to characterize and compare the gene expression profile in KS lesions versus non-cancer tissues from the same individual. Such comparisons could identify pathways critical for KS formation and maintenance. This is the first study that utilized high throughput RNA-seq to characterize the viral and cellular transcriptome in tumor and non-cancer biopsies of African epidemic KS patients. These patients were treated anti-retroviral therapy with undetectable HIV-1 plasma viral load. We found remarkable variability in the viral transcriptome among these patients, with viral latency and immune modulation genes most abundantly expressed. The presence of KSHV also significantly affected the cellular transcriptome profile. Specifically, genes involved in lipid and glucose metabolism disorder pathways were substantially affected. Moreover, infiltration of immune cells into the tumor did not prevent KS formation, suggesting some functional deficits of these cells. Lastly, we found only minimal overlaps between our in vivo cellular transcriptome dataset with those from in vitro studies, reflecting the limitation of in vitro models in representing tumor lesions. These findings could lead to the identification of diagnostic and therapeutic markers for KS, and will provide bases for further mechanistic studies on the functions of both viral and cellular genes that are involved.

## Author summary

Kaposi’s sarcoma-associated herpesvirus (KSHV) is endemic in sub-Saharan Africa and cause Kaposi’s sarcoma (KS). KS is one of the most common cancer among HIV-1 patients in this region. Despite anti-retroviral treatment, prognosis for KS is poor with high mortality often due to presentation of late cancer stage. In order to identify biomarkers or therapeutic targets against KS, a better understanding of the viral and cellular genes expression/transcriptome in the tumor will be necessary. We used RNA-seq, a highly efficient method to sequence transcriptome, to characterize and compare the viral and cellular transcriptome in tumor and non-cancer tissues from KS patients. We found that viral genes involved in latency and immune modulation are most commonly expressed among KS patients. Additionally, cellular genes involved in lipid and glucose metabolism disorder pathways are significantly affected by the presence of KSHV. Despite the detection of immune cells in the tumor, it did not prevent the tumor progression, suggesting some level of immune cell dysfunctions in KS patients. Lastly, we found limited overlap of our data, derived from actual KS biopsy, with other cell culture models, suggesting that the complexity of tumor is difficult to be reflected in cell line models.

## Introduction

Kaposi’s sarcoma (KS) is a multi-focal pleomorphic, highly vascularized tumor that is one of the AIDS defining illnesses. KS mostly manifest as cutaneous lesions, but it can also be detected in the oral mucosa, lymph nodes and all visceral organ systems. There are four types of KS: iatrogenic, classic, endemic and epidemic/AIDS-related KS [1–4]. Endemic and epidemic KS are the most common forms in sub-Sahara Africa. Endemic KS occurs in HIV-1 negative African patients of both genders. The presentation in both genders at varying ages differentiates this form of KS from classical KS found mostly in elderly Mediterranean men. Epidemic KS occurs in patients who are HIV-1 positive, and along with iatrogenic KS, which results from chemical immunosuppression, highlights the role of immune dysregulation in KS development. Depending on the location of lesions, KS patients may display signs and symptoms such as nausea, vomiting, dysphagia and dyspnea. Cutaneous lesions may also disfigure and lead to social stigma for KS patients [5, 6]. The prognosis of KS patients in sub-Sahara Africa is generally poor, often due to delayed diagnosis and late initiation of treatment [7]. Even after receiving anti-retroviral drugs therapy (ART), the mortality of epidemic KS patient is often high due to advanced KS disease staging, simultaneous advanced HIV-1 diagnosis and both HIV-1 and KS-immune reconstitution inflammatory syndromes [8].

The etiologic agent associated with KS is the Kaposi’s sarcoma-associated herpesvirus (KSHV) or human herpesvirus type 8 (HHV-8) which is a member of the gamma-herpesviridae. It is an enveloped virus with a double-stranded viral DNA of approximately 170-Kb. The KSHV genome encodes more than 80 genes that are expressed in a regulated transcriptional program that promotes latency with very limited viral expression, or supports lytic replication with the production of progeny virions. The cellular tropism of KSHV includes epithelial, endothelial, B cells, and more recently has been expanded to include neurons [9–17]. Depending on the host cell environment encountered upon infection, the virus may establish latency and only express latency related genes such as latency-associated nuclear antigen (LANA). LANA expression helps KSHV prevent programmed cell death and circumvent host immune surveillance [18–22]. During reactivation, the entire KSHV genetic repertoire is expressed enabling virus replication and propagation.

KSHV has a high seroprevalence in sub-Sahara Africa, where over 50% of the population has detectable anti-KSHV antibodies [23]. The virus can be transmitted by saliva and sexual routes [24–28]. In addition to KS, KSHV is also the etiological agent of other lymphoproliferative disorders such as multicentric Castleman’s disease (MCD) and primary effusion lymphoma (PEL) [15, 29, 30]. KS can be an opportunistic malignancy commonly presenting in untreated HIV-1 infected patients [31]. Due to the high HIV-1 prevalence in sub-Sahara Africa countries such as Zambia and Tanzania, KS is the leading cancer in men and the third leading cancer in women in Africa [32, 33]. In contrast to the drastic reduction of KS in resource-rich regions, widespread implementation of ART in sub-Saharan Africa, has only modestly reduced KS incidence among the resource-limited countries of the region [34, 35].

There are currently no existing targeted immunotherapies against viral or host proteins in KS, and there is no vaccine against KSHV infection. Indeed the correlates of KS development or protection are ill-defined. A more complete understanding of changes in host and viral gene expression in KS lesions compared with non-tumor tissues could provide insights into the interactions between KSHV and the host that drive development of KS. Moreover, it would allow exploration for any potential biomarkers that are unique to KS neoplastic cells. These biomarkers could make early detection of KS possible and perhaps help to eliminate one of the major malignancies in sub-Saharan Africa. In addition, gene expression profiles of KS lesions may also lead to identification of pathways required for KS neoplasia. Inhibition of such essential pathways could potentially provide therapeutic benefit for KS patients.

RNA-Seq is an extremely high density, unbiased sequencing technique that allows high-throughput assessment of total cellular transcription. It exhibits a broader dynamic range and specificity than microarray analyses [36, 37]. Thus RNA-Seq is an ideal tools for defining the gene expression profiles or transcriptome of KS lesions. RNA-Seq has been utilized in transcriptomic analysis of various forms of cancers to identify pathways that could provide potential targets for future diagnosis or therapy [38, 39]. However, only a limited number of in vitro studies on KSHV infected cell lines or infected monocultures of specific lineages of cells, have utilized either RNA-Seq or microarray for transcriptomic analyses [40–47]. In the current study, epidemic KS lesion biopsies were collected from Zambia and Tanzania for comparative transcriptomic analysis using RNA-Seq. Ipsilateral or contralateral sampling of normal tissues from the same KS patients provided for direct intra-subject comparison of gene expression profiles. We found that KSHV has significantly impacted the cellular gene expression that were involved in the lipid and glucose metabolism disorder pathways. To our knowledge, this is the first study to utilize RNA-Seq to provide unbiased gene expression profile directly from KS lesions in comparison to that of non-neoplastic tissue from the same subject. Our study provides unique insights into the interactions between KSHV and the host and may point to previously unrecognized pathways in KS pathogenesis.

## Materials and methods

### Ethics statement

Permission to conduct this study was obtained from Tanzania National Institute for Medical Research, Ocean Road Cancer Institute Review Board, University of Zambia Biomedical Research Ethics Committee and the Institutional Review Board of the University of Nebraska-Lincoln. Written informed consents were obtained from all study participants and the study did not interfere with the routine clinical care of the participants as per institute’s guidelines.

### Sample collection and processing

Histologically confirmed epidemic KS patients (≥ 18 years old) were recruited from University Teaching Hospital (UTH), Zambia and Ocean Road Cancer Institute (ORCI), Tanzania. Whole blood was collected from each patient and separated into plasma and mononuclear cells using Lymphoprep according to the manufacturer’s protocol (Stemcell technologies, Massachusetts, USA).

A 4 mm cylindrical single-use punch was used to collect biopsy samples from a representative KS lesion and a second identical punch biopsy was collected from an uninvolved ipsilateral /contralateral site on the same individual. The collected tissues were treated overnight in RNALater (Ambion) to facilitate subsequent DNA/RNA extraction. RNALater was removed prior to freezing at -80°C.

### Determination of HIV-1 status and plasma viral load

The HIV-1 status for the Tanzanian KS patient was determined using the Tanzania HIV Rapid Test Algorithm. Zambian KS patients’ HIV-1 status was determined using the Alere Determine HIV-1/2 Ag/Ab Combo test. The HIV-1 serostatus of all subjects was re-confirmed at the University of Nebraska-Lincoln with the HIV-1-2.0 First Response kit (Premier Medical Corporation Limited, Daman, India).

HIV-1 viral RNA was extracted from plasma using the QIAamp viral RNA mini kit following the manufacturer’s protocol (Qiagen, Hildren, Germany). HIV-1 plasma viral load was quantified by RT-PCR (QuantStudio 3, Applied Biosystems, Carlsbad, CA) using oligonucleotides HIV-1 LTR forward primer [5’-GCCTCAATAAAGCTTGCCTTGA-3’], reverse primer [5’- GGGCGCCACTGCTAGAGA-3’] and probe [5’-FAM-CCAGAGTCACACAACAGACGGGCACA-BHQ_1–3’] under the following reaction conditions: 50°C for 15 min, 95°C for 2 min; 45 cycles at 95°C for 15 sec and 60°C for 30 sec.

### Detection of KSHV DNA in plasma

KSHV DNA was extracted from the patient plasma with QIAmp DNA mini kit (Qiagen, Hilden, Germany) according to manufacturer’s protocol. The extracted DNA was confirmed to be PCR negative for β-actin to demonstrate the absence of cellular DNA contamination. To detect the presence of KSHV DNA, nested-PCR for KSHV ORF26 was performed on the extracted DNA (1^st^ round PCR primers: forward [5’-AGCCGAAAGATTCCACCAT-3’] and reverse [5’-TCCGTGTTGTCTACGTCCAG-3’], 2^nd^ round PCR primers: Forward [5’-CGAATCCAACGGATTTGACCTC-3’] and reverse [5’-CCCATAAATGACACATTGGTGGTA-3’]) using the following conditions: 95°C for 5 min; 35 cycles at 95°C for 30 sec, 58°C for 30 sec, 72°C for 30 sec; 72°C for 7 min. The final PCR product was analyzed on a 1% TAE agarose gel versus a KSHV ORF26 DNA positive amplification product at 173 base pairs. Genomic DNA from the KSHV chronically infected BC3 cell line was used as positive control.

### Quantification of KSHV DNA load in lesion tissues

Fresh frozen biopsies from KS lesions were homogenized by cryo-cracking with liquid nitrogen. Genomic DNA was extracted from the homogenized sample using Puregene genomic DNA purification kit (Qiagen, Hilden, Germany) following the manufacturer’s protocol. To quantify the KSHV DNA load from the lesion, the extracted DNA was analyzed by real-time PCR against KSHV ORF26 with the 2^nd^ round PCR primers set listed above and the following probe [5’-FAM-CCATGGTCGTGCCGCACGCA-BHQ_1–3’]. A plasmid encoding KSHV ORF26 was used to create a standard curve to compare patient DNA amplification signals. The number of cells analyzed was quantified by comparing patient DNAs to a standard curve produced by real-time PCR for the β-globin gene. Genomic DNA from the human cell line 8E5 was used as a standard. All reactions were performed in triplicate under the following conditions: 50°C for 2 min, 95°C for 10 min; 40 cycles of 95°C for 15 sec, and 60°C for 1 min.

### RNA library preparation and RNA-Seq

Fresh frozen biopsies from KS lesions and ipsilateral/contralateral normal sites were homogenized by cryo-cracking in liquid nitrogen using a mortar and pestle. Briefly, the mortar and pestle were cleaned and chilled in a metal tray with liquid nitrogen. The sample was transferred from -80°C storage on dry ice into the pre-chilled mortar, followed by pulverize and homogenize using the pre-chilled pestle. The homogenized sample is then transferred into a 2-ml Eppendorf tube by a pre-chilled spatula for genomic RNA extraction.

Genomic RNA was extracted from the homogenized sample with miRNeasy mini kit with on-column DNase I treatment according to the manufacturer’s protocol (Qiagen, Hilden, Germany). Concentration of the extracted RNA was measured with a Qubit fluorometer using the Qubit RNA Broad-Range kit (Invitrogen, Waltham, MA). Quality of the extracted RNA was assessed by an Agilent Bio-Analyzer system (Agilent Technologies, Santa Clara, CA) for fragment analysis. By measuring the 18s and 28s rRNA peaks within the electrophoretic trace, the RNA Integrity Number (RIN) was calculated to determine the level of RNA integrity. Only samples with an RIN >4.9 were used for library preparation and RNA-Seq. Library preparation was performed using the TruSeq^™^ RNA Library Prep Kit v2, and RNA-Seq data were collected using an Illumina HiSeq2500 in single-read, 50 bp Rapid Run mode at the University of Nebraska Medical Center DNA Sequencing Core.

### RNA-Seq data analysis

The RNA-seq data was aligned against human (hg19 genome, Ensemble v75 transcriptome) or KSHV (NC_009333) genomes and transcriptomes using the bowtie2 algorithm, and RSEM v1.2.31 software was used to estimate read counts and FPKM values at the gene level [48, 49]. Raw counts were converted to log2 (5+count) values and quantile normalized to use in Principle Component Analysis and gene expression heatmaps. Significance and fold change of differential expression between lesion and control samples was estimated using the DESeq2 method on raw values and genes with false discovery rate (FDR) <5% were considered as significant [50]. An additional threshold of 5-fold was used to enumerate a set of most-changed genes between conditions. Pearson correlation was used to test associations between KSHV transcript loads in tissues for significantly differentially expressed genes. Correlations with p<0.05 were considered significant.

KSHV gene functional definitions were obtained from Arias et.al. [51]. Hierarchical clustering based on the KSHV gene expression was done on the transcripts with at least 10 counts in at least one sample using Spearman correlation distance for genes and Euclidean distance for patients using average linkage. The final gene clusters were defined using a distance threshold of 0.5. Functions were then assigned for each cluster based on the function with the best enrichment ratio E = % genes in cluster / % genes total. Hierarchical clustering and principal component analysis was done on z-score converted normalized values using MATLAB R2016a (v9.0.0). Expression heatmaps were plotted in Microsoft Excel using normalized values centered versus average across all samples.

Gene set enrichment analysis was done using Qiagen’s Ingenuity Pathway Analysis software (IPA, QIAGEN Redwood City,www.qiagen.com/ingenuity) using “Upstream Analysis”, “Regulator Effects” and “Diseases & Functions” options. Activation Z-scores (Z), calculated by IPA are a result of combining mRNA expression changes from the experiment and known effect of the gene on function or upstream regulator on the target (e.g every downregulated gene suppressing a function or upregulated gene activating a function will contribute to predictions as to whether the function is activated). |Z|>2 predictions were considered significant. Upstream regulators that were significantly differentially expressed at the mRNA level, had significantly overrepresented number of known targets (p<10^−5^) and had a significantly predicted activation state (|Z|>2) were considered. The only regulator effect network with consistency score >100 is reported (the next best network had a score of 43). Functions that passed p<10^−15^ threshold or P<10^−5^ and |Z|>2 threshold were considered.

For analyzing relationships between genes involved in the lipid and glucose models, Ingenuity Knowledgebase was used to derive information for all considered functions. A combined model was built to demonstrate two aspects: overlap between lipid-related functions, and the cross-talk of lipid functions with glucose metabolism disorder. Functions that shared 100% of genes with another function were omitted (e.g. all genes from metabolism and synthesis of triacylglycerol were in metabolism of acylglycerol and all genes from concentration of acylglycerol were in concentration of lipid). Three of the other 6 lipid-related functions had most, except for 1 or 2 (>90%) genes as members of a more general function and are depicted in the model as sub-functions. Information about genes’ effect on each function was considered and the potential cross-talk is annotated. Genes that did not have a consensus effect on any of the functions received an “affects” designation. If a gene was known to increase or decrease activity of any function, it was assigned to one or to both effect categories in cases of both increase and decrease calls. Additional information includes number of genes specific to the particular function (not involved in any other considered functions) and the overall number of genes from the 3 effect categories for all major functions.

CIBERSORT software predictions were based on gene FPKM values using the LM22 cell signature model in “absolute” mode [52]. Cell abundance was then compared using a two-sample t-test. Significance was defined at p<0.05.

RNA-seq data from external GEO sets (GSE62829, GSE62344, GSE84237) were processed as described above and tested for differential gene expression using DESeq2. Microarray data from external sets (GSE6489, GSE45590, GSE66682) was quantile normalized and log_2_-transformed before statistical evaluation by one-way ANOVA with correction for multiple testing to estimate FDR as previously described [53].

The original RNA-seq data from this study was uploaded to the GEO database (https://www.ncbi.nlm.nih.gov/geo/) with the accession number GSE100684.

### RNA-seq validation with real time-PCR

To verify the RNA transcript level changes observed from the transcriptomic profiling data, a two-step qRT-PCR methods was used to measure the RNA transcript level for selected host genes. First, DNase I-treated total RNA extracted from the tissues was subjected to cDNA synthesis using Superscript III reverse transcriptase primed with random 9-nonamers (Invitrogen, Waltham, MA). The amount of cDNA was measured with Qubit ssDNA assay kit (Invitrogen, Waltham, MA). Real-time PCR reactions were prepared using iQ^™^ SYBR Green supermix (Biorad, Hercules, CA). Each PCR reaction contained 20 ng of cDNA with primer pairs for the respective host genes (S1 Table). qRT-PCR was performed in a QuantStudio3 instrument (Applied Biosystems, Waltham, MA) under the following cycling parameters: 50°C for 2 min, 95°C for 10 min; 40 cycles at 95°C for 15 sec and 60°C for 1 min; followed by melt-curve parameters: 95°C for 15 sec, 60°C for 1 min, 95°C for 15 sec. Lesion tissues were measured in triplicate, while normal tissues were measured in duplicate due to reduced cDNA yield. Glyceraldehyde 3-phosphate dehydrogenase (GAPDH) was used as an internal standard for each target gene. The RNA transcript level was presented as relative quantification (RQ) using the comparative cycle threshold (ΔΔCT) method. Briefly, the ΔCT for each target gene was calculated by subtracting the CT for GAPDH in the same sample from the CT for target gene.

For each target gene, their respective average ΔCT from the normal tissue was used as a reference for the calculation of ΔΔCT by subtracting ΔCT for normal tissue from the ΔCT for lesion tissue. Finally, the RQ for each target gene is defined as 2^- ΔΔCT^.

## Results

### Demographic of epidemic KS patients

Four male HIV-1 positive epidemic KS patients 37 to 54 years of age were recruited from Zambia and Tanzania. Two of the four patients (p32 and p83) had been on ART for over 2 years at the time of inclusion in the study. The other patients (p22 and p23) had received ART for 2 to 3 months (Table 1). All patients were fully suppressed with undetectable HIV-1 plasma viral load. However, KSHV viral DNA was detected in the plasma of three out of four patients. Quantification of KSHV load in the extracted lesion tissue DNA revealed 1.14x10^5^ and 8.17x10^5^ copies/million cells in the long-term ART treated patients (p32 and p83 respectively), while the recently ART treated patients (p22 and p23) had a lower KSHV load at 6.93x10^3^ and 5.44x10^4^ copies/million cells. The extracted RNAs from lesions and control tissues were used for subsequent RNA-seq analysis.

**Table 1:** Patient demographic information.

Patient ID	Country	Sex	Age	HIV status	ART duration (months)	Plasma HIV load	Plasma KSHV load	Lesion KSHV DNA load (copies/10^6^ cells)
p22	Zambia	M	46	+	3	-	+	5.44 x 10^4^
p23	Zambia	M	40	+	2	-	+	6.93 x 10^3^
p32	Zambia	M	37	+	60	-	+	1.14 x 10^5^
p83	Tanzania	M	54	+	24	-	-	8.17 x 10^5^

Legends: M = Male; + = detectable;— = undetectable

### RNA-seq and analysis

**Fig 1:**
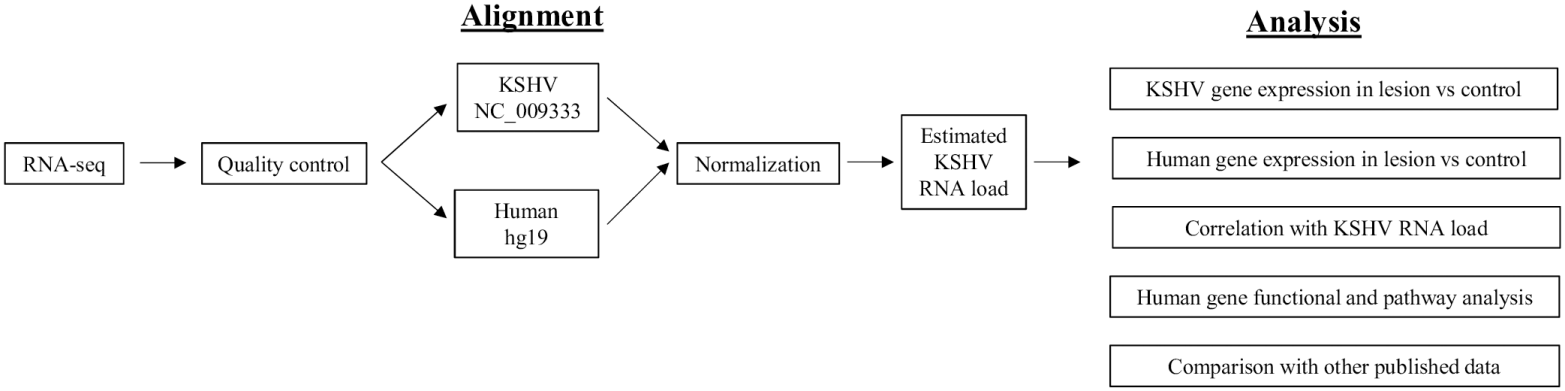
Schematic of RNA-seq analysis work flow.

The RNA extracted from the lesion and control tissues were sequenced at a depth of over 12 million reads/sample (Table 2). After passing the standard quality control, reads with good quality scores, were aligned against the reference human and KSHV genomes and transcriptomes (Fig 1). The majority of reads aligned to the human transcriptome, with at least 79% and 72% alignment rate for the lesion and control tissues, respectively. As expected, there were more reads aligned to the KSHV transcriptome in KS lesions, (range 718–17202 reads) compared to control tissues (range 0–60 reads) (Table 2). Although low KSHV transcripts reads were detected in some control tissues, it represent less than 2% of the viral reads detected in the corresponding KS lesion and likely originated from the KSHV positive circulating peripheral blood mononuclear cells (PBMC). After normalization to the total number of aligned reads, the KSHV RNA load within each tissue was estimated from the total read count aligned to the KSHV transcriptome. Gene expression profiles for both human and KSHV transcriptome were then determined for both the lesion and control tissues, as well as in correlation with the KSHV RNA load in the lesion. Genes that were demonstrated to be significantly up or downregulated were then selected for upstream regulator, functional and pathway analyses to explore potential mechanisms dysregulated by KSHV in KS lesions. Finally, genes that were differentially expressed in the lesions were comparatively overlapped with other published KSHV microarray or RNA-seq datasets.

**Table 2:** RNA-seq raw reads and alignment statistics.

Class	Patient ID	Total reads	Aligned Human Transcriptome	Aligned KSHV Transcriptome
**Lesion**	p22	15,471,116	12,849,035 (83%)	1,650
	p23	12,060,351	10,225,520 (85%)	17,202
	p32	15,907,935	12,629,009 (79%)	718
	p83	15,947,529	13,198,922 (83%)	3,441
**Control**	p22	13,818,393	10,543,129 (76%)	2
	p23	14,040,685	10,745,704 (77%)	0
	p32	14,307,950	10,240,092 (72%)	0
	p83	14,653,312	10,872,584 (74%)	60

### KSHV gene expression profile diversity in individual lesions

**Fig 2:**
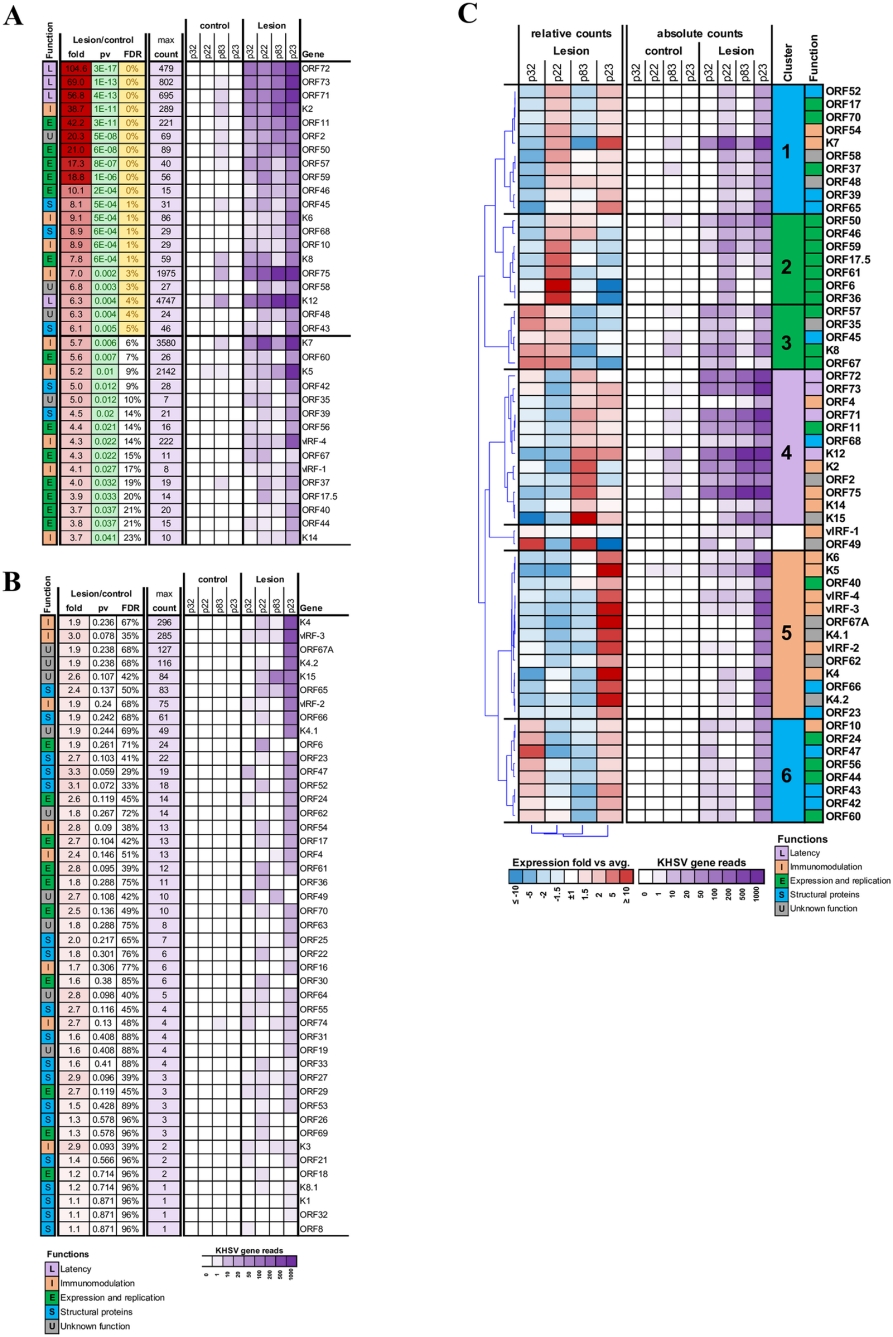
Expression and functional classification of KSHV genes. (A) KSHV genes significantly upregulated in the lesions (p<0.05). (B) Other KSHV genes detected in the lesions. (C) Clustering of genes and patients based on KSHV genes with ≥10 reads in at least one sample. Each cluster with ≥5 genes was then annotated with the most enriched function.

Alignment of the KSHV gene expression profile in the lesion and control tissues to the reference KSHV genome and transcriptome showed that nearly no KSHV expression was detected in the control tissues, except in patient p83, where 60 reads in the control tissue aligned to the KSHV transcriptome (Fig 2A). In contrast, robust KSHV gene expression was readily detected in all lesion tissues. Twenty viral genes were expressed at levels ≥ 6-fold than in the control tissues. These differences were statistically significant with a false discovery rate (FDR) of ≤ 5% with additional 15 genes passing nominal *p* ≤ 0.05 (Fig 2A). Other viral genes were also detected in the lesion tissues, but differentials did not reach significance (Fig 2B). Lack of statistical significance is a result of variant levels of expression of these viral genes among the four lesion samples. For example, K5 and vIRF3 gene expression was clearly highly upregulated in the lesion tissue of patient p23 compare to its corresponding control tissue, yet the significance of this difference is masked by the relatively low expression of the same gene in lesions from the other three patients.

Next, we explored how the differences in viral gene expressions relate to their known or presumed functions. Viral genes were classified based on the functional categories of latency, expression and replication, structural proteins, immunomodulation and undefined functions. Normalized expression of genes showing at least 10 aligned reads was used for hierarchical clustering. The analysis yielded 6 major clusters of genes showing distinct patterns among the four patients (Fig 2C). While all lesions tissues expressed latency genes at high levels, patients p83 and p23 had relatively higher expression of those genes (cluster 4). Relative expression in the lesion tissues from patients p22 and p23 was higher for genes from the expression and replication categories (clusters 2, 3). KSHV structural clusters was defined by upregulation in patients p22 and p23 (cluster 1) and patients p23 and p32 (cluster 6), suggesting a higher level of lytic replication in tissue. Immunomodulation related genes were expressed in all patients, with patient p23 displaying the highest expression level (cluster 5).

### Parallel human gene expression profile among epidemic KS lesions

**Fig 3:**
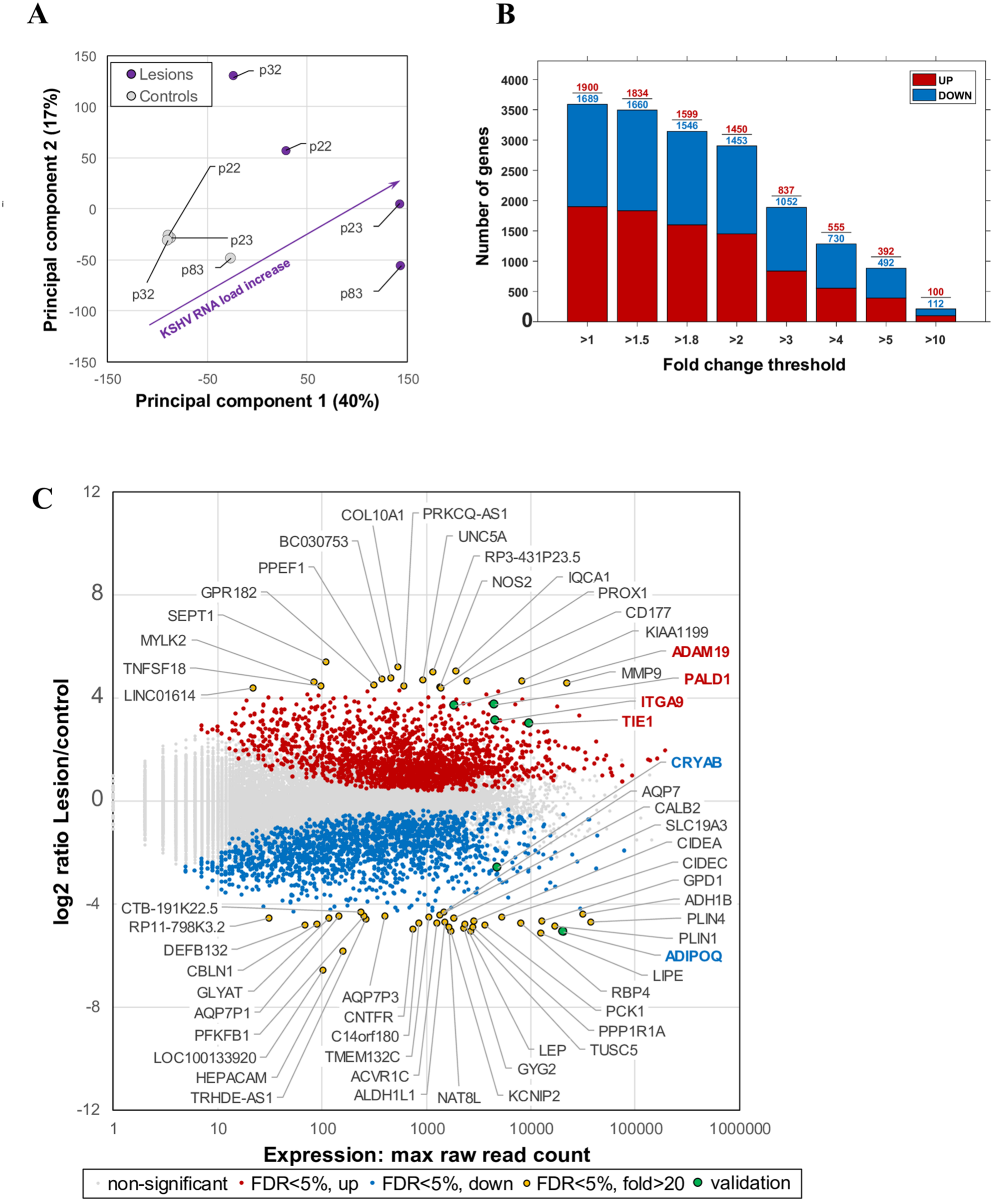
Cellular gene expression. (A) Relationship between the samples based on cellular gene expression using the Principal Component (PC) analysis. Combination of the first and the second PC coincided with the KSHV RNA load, indicating that KSHV genes expression affected the cellular gene expression. (B) Number of cellular genes significantly (FDR<5%) differentially upregulated (red) or downregulated (blue) in the lesion vs control samples, expressed in fold changes. (C) Significantly differentially expressed cellular genes and their raw expression (maximum raw read count across samples). All cellular genes with ≥20 fold change are highlighted with yellow dots and genes selected for real time-PCR validation are highlighted with green dots.

After alignment to human transcriptome, relationships between sample were studied by Principle Component Analysis which indicated 40% and 17% sample variability (the first and second principal component, respectively) (Fig 3A). We note that the first principal component mainly corresponds to the KSHV RNA load within the tissue. While the lesion and control tissues formed distinct clusters, the control tissues shows less variability. In addition, control tissue from patient p83 is unique as it associates more closely to the lesion tissues. This association is likely due to the presence of low KSHV RNA level in this control tissue, resulting in marginally similar transcriptional profile to those of lesion samples.

**Fig 4:**
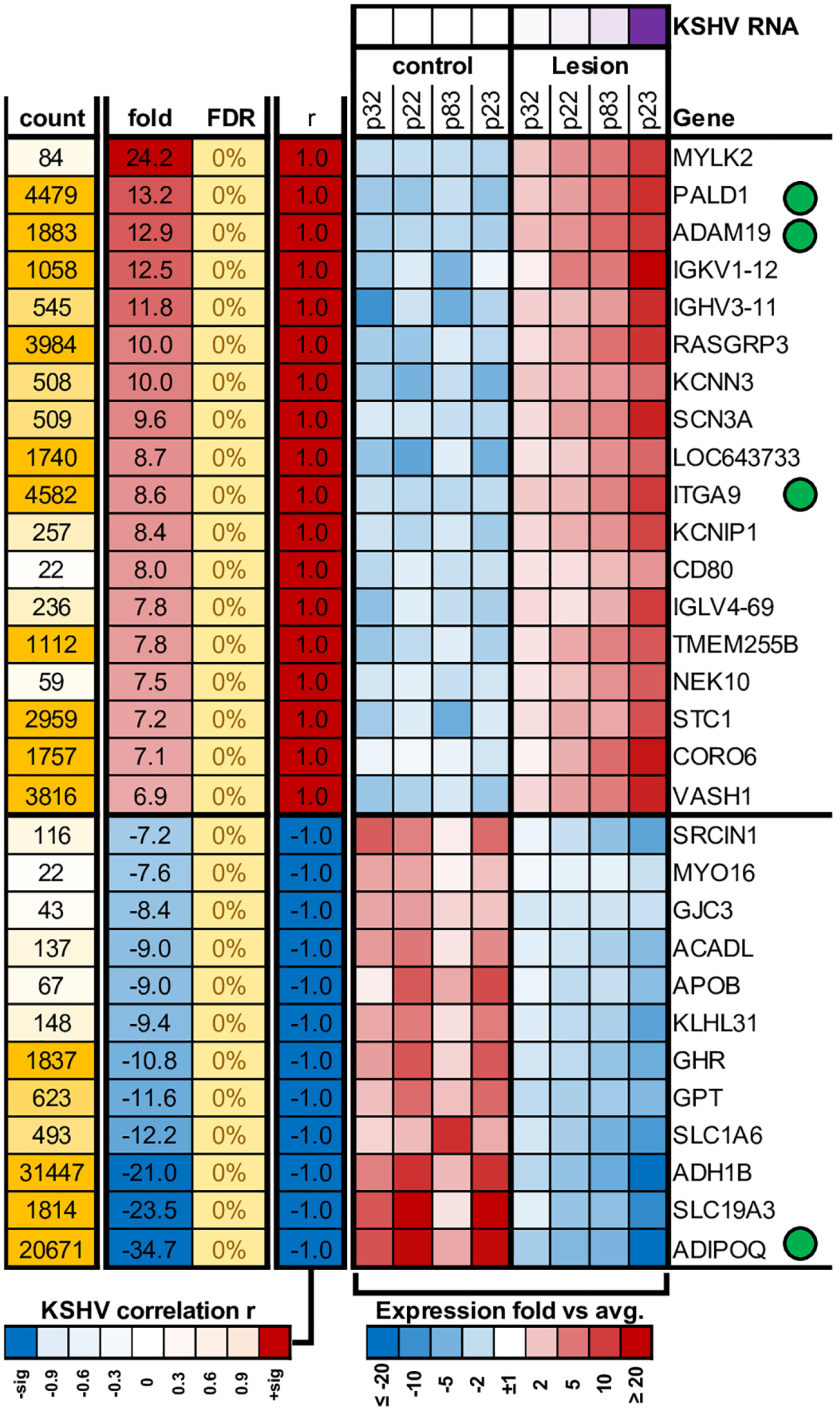
Heat map showing the relative expression of cellular genes with the highest fold changes between the lesion and controls. Top 30 most differentially expressed cellular genes that were also significantly correlated with the KSHV RNA load in the lesions. Green circle indicates genes selected for real time-PCR validation.

Direct comparison of gene expression between the collective lesion and control samples, revealed 3589 significantly differentially expressed genes (FDR < 5%), 1096 of which have ≥ 5 fold changes (Fig 3B) and 52 genes showing ≥ 20-fold change (Fig 3C). A comparison of gene expressions among the 4 lesion/control tissue pairs is shown in S2 Table, demonstrating the highly similar expression pattern among the samples. Expression heatmap for the top 30 known most changed genes, regardless of their correlation with KSHV RNA load within the tissues, are shown for each tissue sample in S1 Fig. In addition, 311 genes in the lesion tissues were both significantly differentially expressed (FDR < 5%) and directly correlated with the KSHV RNA load within the tissues (*p* < 0.05) with top 30 genes shown in Fig 4.

### Verification of cellular gene expression by real time-PCR

**Fig 5:**
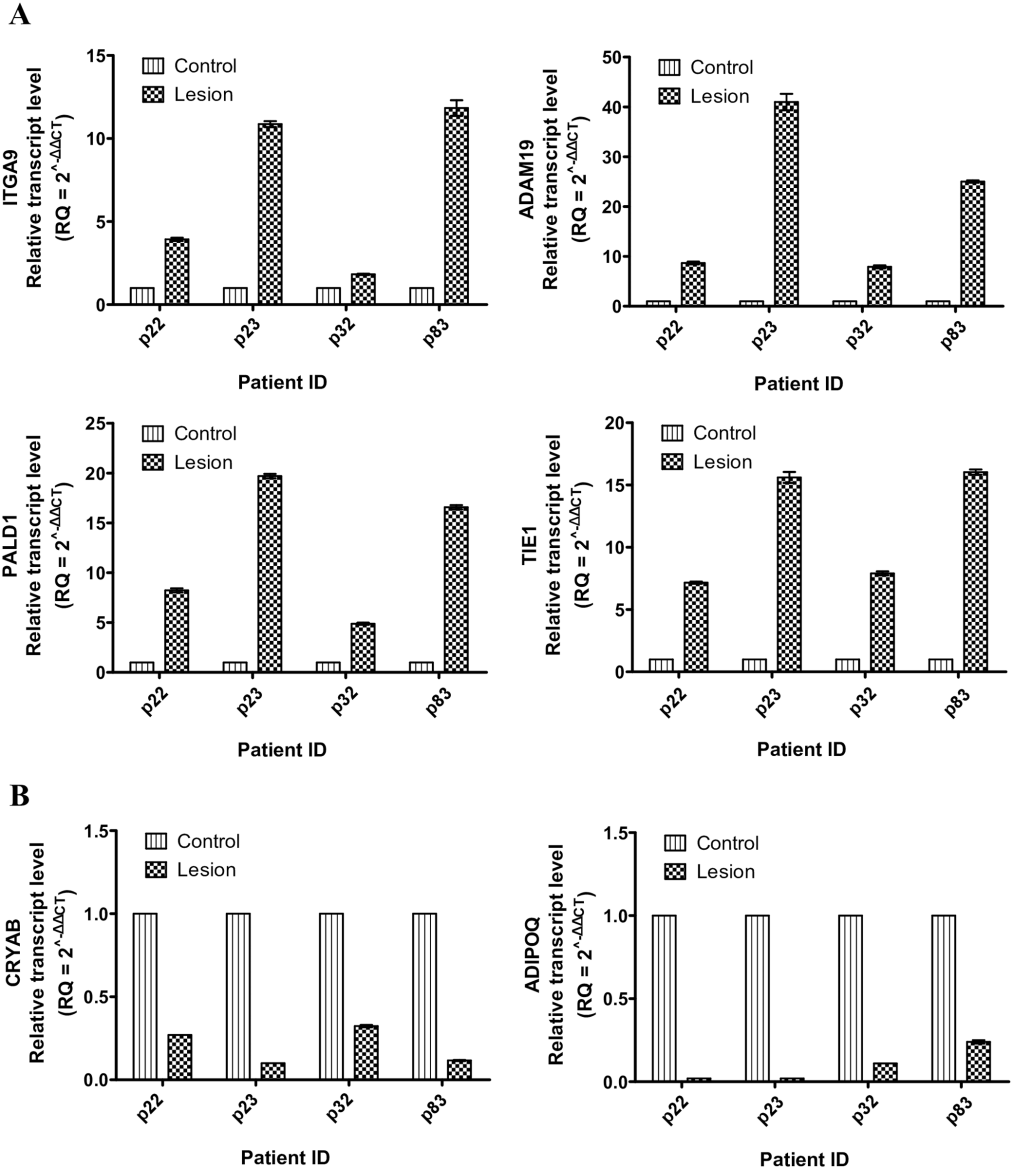
Validation of selected cellular gene expression by real time-PCR. (A) Upregulated genes, ITGA9, ADAM19, PALD1 and TIE1 in the lesion relative to the control tissues. (B) Downregulated genes, CRYAB and ADIPOQ in the lesion relative to the control tissues.

A subset of the gene expression results determined by the RNA-seq analysis were validated by real-time PCR. Six cellular genes, 4 upregulated and 2 downregulated genes, from the lesion tissues were selected. These genes were distributed across the range of expression changes between lesion and control tissues and each target was strongly correlated with KSHV RNA load in the tissues. The up or downregulation expression of these genes as quantified by real time-PCR was concordant with the results obtained through RNA-seq analysis. Patient p23 displayed the highest fold increases in the expression of the 4 upregulated genes examined (Fig 5A). A similar pattern was observed among the downregulated genes, where the patient p23 has evinced the highest fold decreases (Fig 5B). Together, these results confirmed the validity of the gene expression profiles determined from RNA-seq analysis.

### Predicted transcriptional regulator network in KS lesions

**Fig 6:**
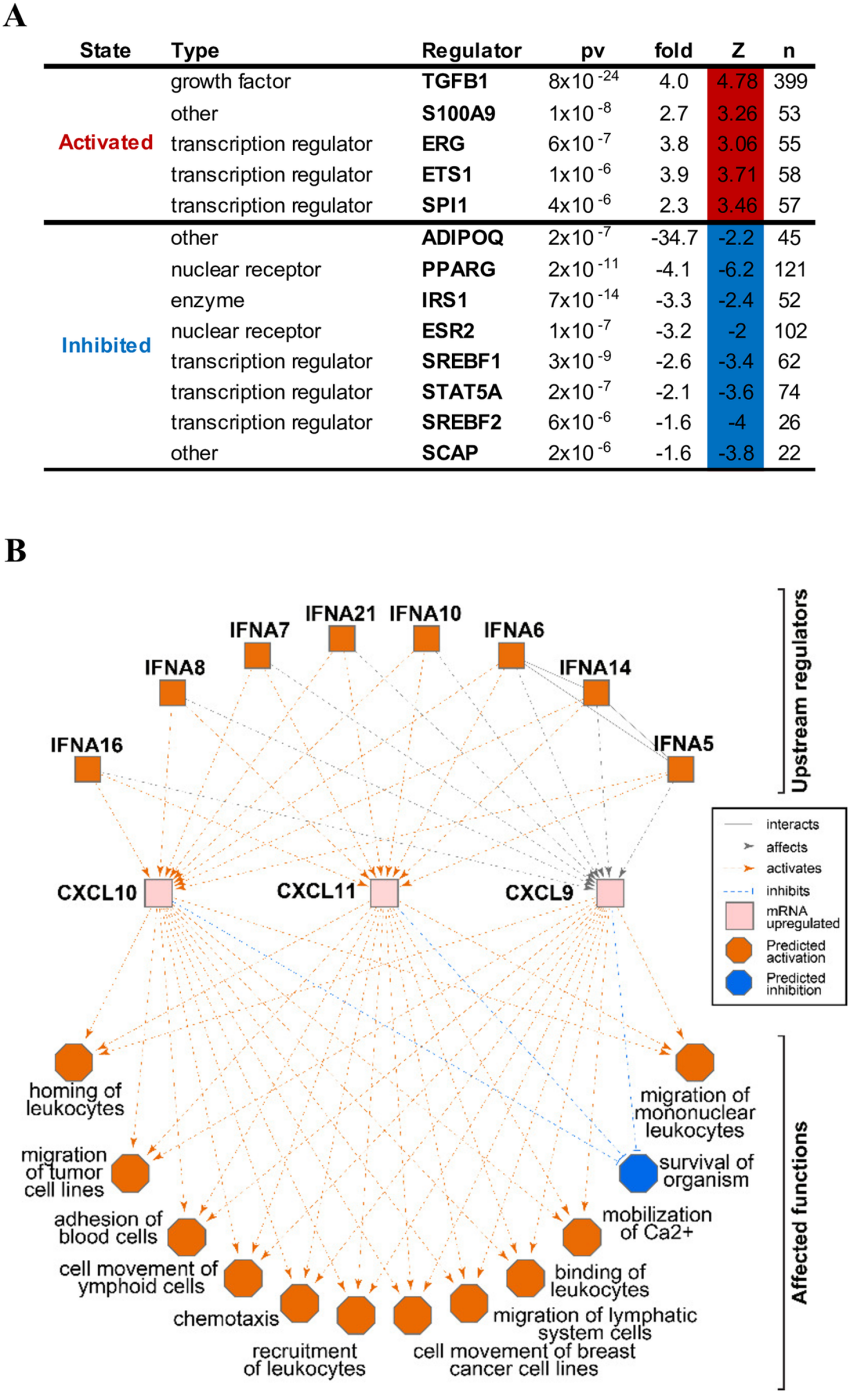
Upstream regulators significantly affected in the lesions. (A) List of known regulators that demonstrated significant expression changes and affected the expression of its known downstream target genes. “pv” = p-value, “n” = number of known target genes changed in lesions, “fold” = expression changes for the regulator, “Z” = z-score for the predicted change of the regulator activity. (B) Top regulator network discovered by Ingenuity Pathway analysis with IFNA predicted to be activated, resulting in expression changes of the CXCL9, 10 and 11 that mainly activate multiple blood-related functions.

Given the profound changes in gene expression profiles observed in KS tissues, the levels of transcriptional regulators for these genes may have been altered and could impact downstream biological processes. To investigate this issue, we tested the set of genes that were significantly differentially expressed in the lesions for enrichment of known key transcriptional regulators using the Upstream Regulator Analysis within the Ingenuity Pathway Analysis (IPA). IPA Knowledgebase contains information about known regulators targets and can predict which transcriptional regulators are responsible for certain biological processes or it can suggest pathways and the potential impact regulators may have on those pathways. Our analysis identified 5 genes upregulated in lesion tissues with a significant number of their targets (p<10^−5^) changed in a direction which indicates an activated regulator status on a protein level (positive Z-score >2) (Fig 6A). The top activated regulator was the tumor growth factor beta-1 (TGFB1), which potentially regulates the expression or function of 399 different genes and plays an important role in various immune responses such as regulation of B cells [54]. Another 8 potential regulators with decreased expression showed a negative Z-score, indicating an inhibited status (Fig 6A). The most inhibited regulator was adiponectin (ADIPOQ), which affects 45 different genes involved in various metabolic pathways.

In order to find other regulators that may impact downstream pathways, we screened the data set with the IPA Regulator Effects analysis. The top scored regulator effect network was the interferon alpha network that drives activation of blood-cell related functions, such as cellular adhesion, extravasation and recruitment of leukocytes, suggesting tissues are producing signals to promote increased infiltration of immune cells into the lesions (Fig 6B).

### Gene expression profile associations with disease and biological function

**Fig 7:**
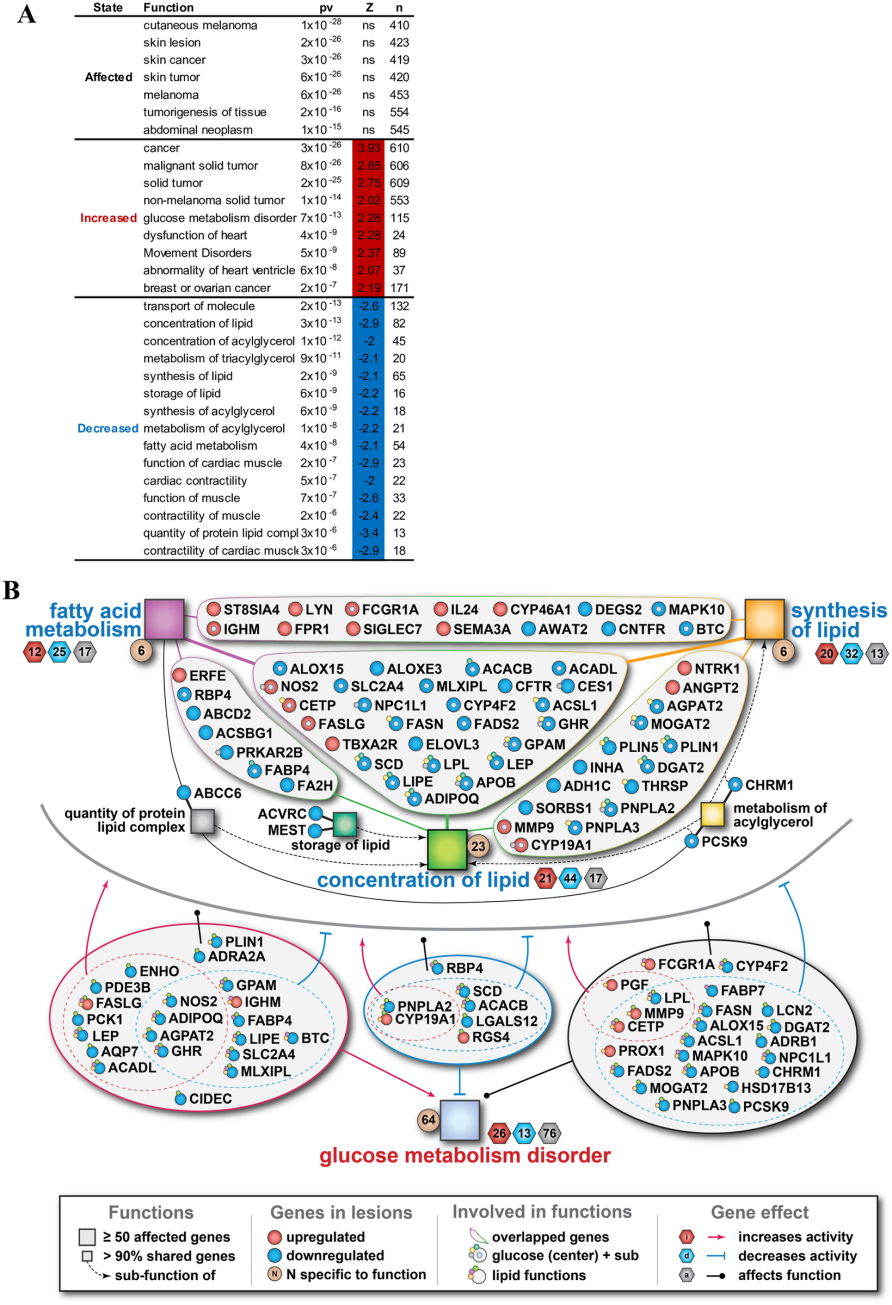
Predicted disease and biological functions associated with cellular gene expression. (A) Enrichment analysis for functions significantly affected in the lesions based on ≥5 fold of gene expression changes. Top functions with significantly predicted activation state (p<10^−5^, |Z|>2) are shown. “pv” = p-value, “n” = number of known target genes changed in lesions, “Z” = z-score for the predicted change of activity, “ns” = unassigned. (B) Significantly changed genes involved in overlapping lipid-related functions and/or glucose metabolism disorder in lesions are shown. Small squares indicate significantly enriched sub-functions that share >90% of genes with major functions (big squares). Top portion of the figure (above the grey line) represent overlap between various lipid functions, while the bottom portion (below the grey line) illustrates cross-talk with glucose metabolism disorder. The hexagons beside major functions indicate the number of genes contributing to that function’s activation (red), inhibition (blue) or unspecified effect (gray). The brown circles beside major functions indicate the number of genes which belong exclusively to that major function. Circles indicate genes shared between at least 2 functions that are up-regulated (red) or down-regulated (blue). Gene involvements are shown by solid and dotted lines (red for increased activity, blue for decreased activity, black for unspecific activity) and by small circles attached to a given gene with color matched to the color of square representing the corresponding function. *Illustrative example*: Concentration of lipid (green big square) in the center of the figure is repressed in lesion (blue font), as evidenced by 44 genes suppressing that function in lesions (blue hexagon), while only 21 genes activate the function (red hexagon) and 17 genes affect it in an unspecified direction (gray hexagon). Out of these 82 genes, there are 23 unique genes (brown circle) specific to this function and the rest are shared with at least one other function. Green line link with the upper left group of genes that are shared with fatty acid metabolism. Green line link with the upper right group of genes that are shared with synthesis of lipid. Green line in the middle link to the group of genes that are shared between the three major lipid functions (Fatty acid metabolism, concentration of lipid and synthesis of lipid). ADIPOQ (locates at the bottom of the middle group of genes) is a gene shared between these three major lipid functions. It is also involved in the metabolism of acylglycerol (attached small yellow circle), storage of lipid (attached small dark green circle) and glucose metabolism disorder (center small cyan circle). Given its involvement in glucose metabolism disorder, ADIPOQ is also shown in the bottom portion of the figure where the attached small circles illustrate its involvement in the three major lipid functions. Its location in the red group of genes at the lower left is indicative of genes whose changes result in activation of the function (red arrow). Given that ADIPOQ is represented by a blue circle (down-regulated genes in lesions), it means that the downregulation of ADIPOQ in lesion results in activation of glucose metabolism disorder. Finally, ADIPOQ is among the genes that suppress one lipid function (blue dotted line with ┬) and activate another (red dotted line with arrow).

To better understand which diseases or biological functions were the most correlated with the observed gene expression profiles, we focused our analysis on 884 genes that have at least 5-fold expression changes in the lesion relative to control tissue. As indicated by their p-values, the top 7 functions impacted by KS altered gene expression were all related to cancer. However, we cannot determine if these functions were increased or decreased due to lack of information in the IPA (Fig 7A). More importantly, 4 functions that showed increased activities (Z-Score > 2) were closely associated with cancer (Fig 7A). In addition, another 115 genes associated with glucose metabolism disorder had increased activities (*p* = 7x10^-13^, Z-Score = 2.28). Among the significantly enriched functions that showed decreased activities in the lesion tissues, 8 lipid metabolism functions and 7 functions related to small molecule biochemistry were revealed. These analysis suggested that fundamental glucose and lipid metabolic pathways are substantially altered in the KS lesions.

To further illuminate the relationship of genes involved in the lipid and glucose metabolism in the lesion, the Ingenuity Knowledgebase was used to derive information for all considered functions. Significant downregulation of 49 of 67 genes (73%) with overlap between the 6 lipids functions was noted (Fig 7B, S3 Table). In contrast, the downregulation of genes involved in the glucose pathway associate with increased activity of glucose metabolism disorder, which in turn, could both increase or decrease the activity of the lipid metabolism.

### Cell infiltration into lesion tissues

**Fig 8:**
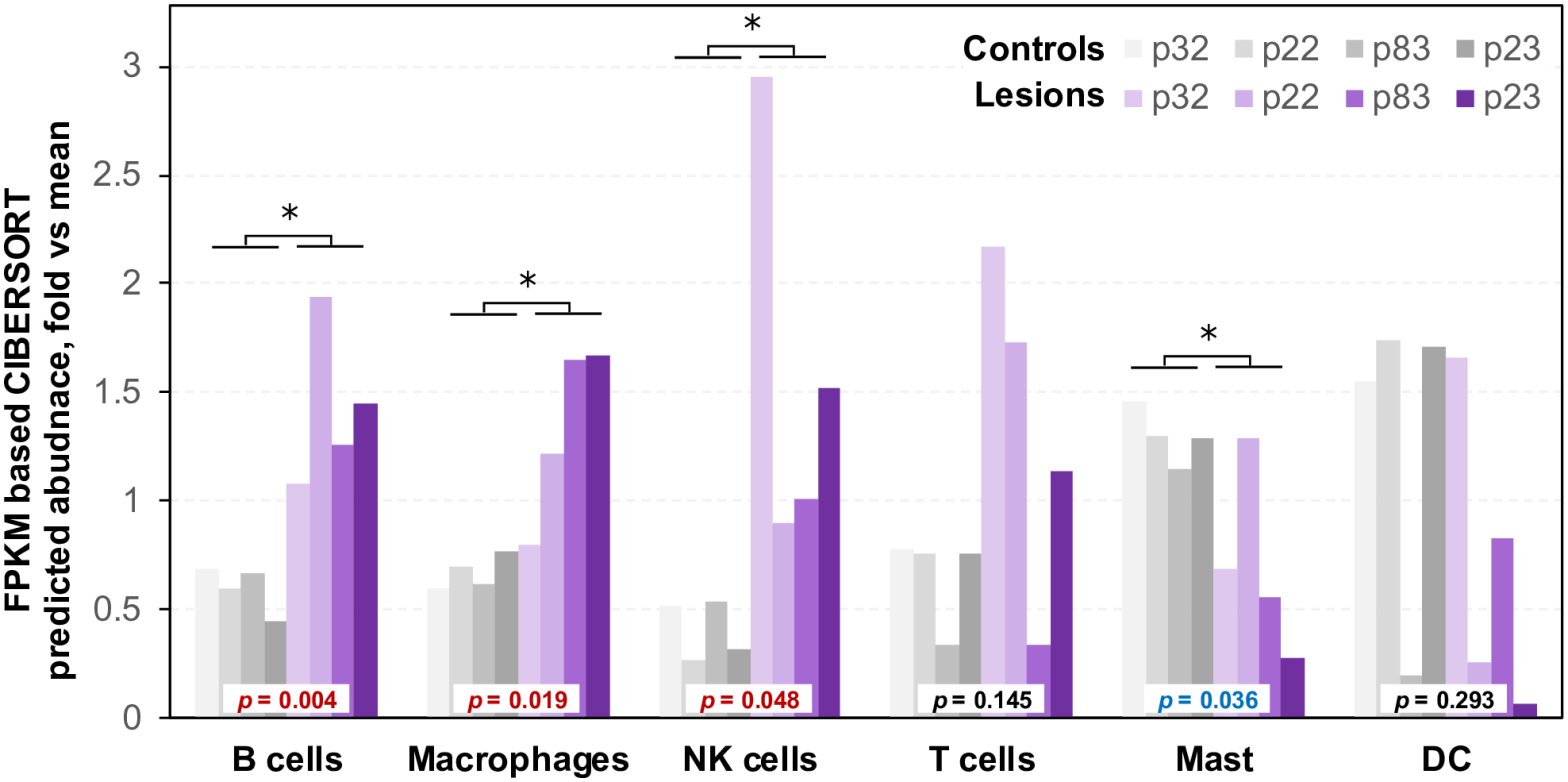
Estimated differences in the blood cell populations between the lesion and control tissues. Blood cell abundance was estimated by CIBERSORT from the gene expression and compared between the lesion and control tissues. Significant p-values are highlighted in red for the increased and in blue for the decreased blood cell abundance in the lesion. * denote statistical significant in comparison to the controls.

One of the roles for immune cells is to surveil against foreign antigens, however, such surveillance, if present, clearly did not prevent the development of KS lesions in these evaluated cases. To test the hypothesis that failure of immune cells to target and infiltrate tumor tissue contributed to KS, we interrogated the cellular gene expression dataset with the CIBERSORT software to estimate the proportions of immune cells in tissues. We found that the levels of B cells, macrophages and NK cells in lesions were predicted to be significantly higher than in control tissues (*p* = 0.004, *p* = 0.019 and *p* = 0.048, respectively), and mast cells were lower (*p* = 0.036) (Fig 8). T cell infiltration into the lesions was only predicted in 3 of 4 patients, and gene signatures associated with dendritic cell infiltration was only detected in lesions from patients p32 and p83, but were not higher than in control tissues. These results are congruent with the regulator effect prediction above that suggested KS tumors were producing chemo-attractants to promote infiltration of immune cells.

### Similarity between in-vitro studies and actual lesion tissues

**Fig 9:**
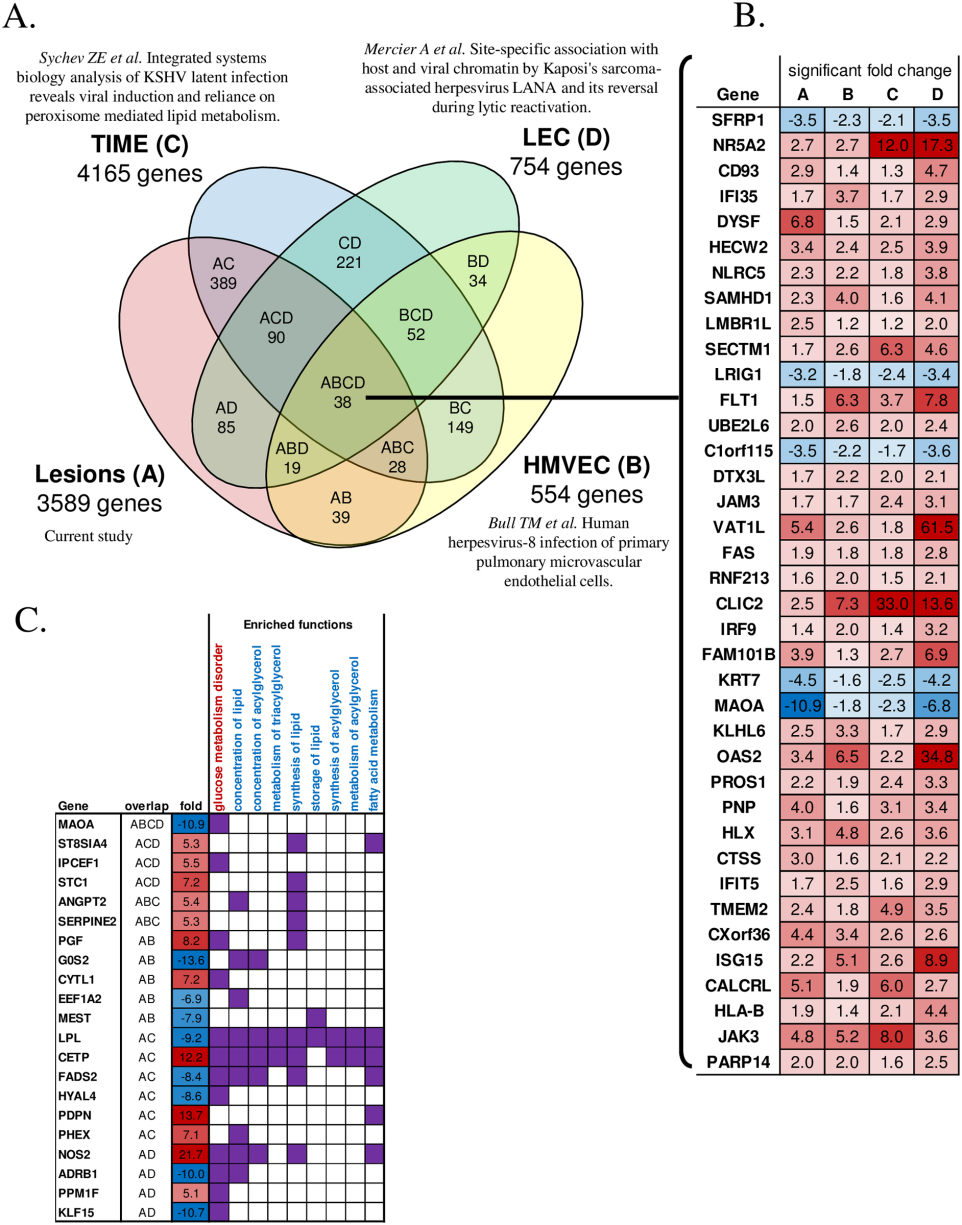
Gene overlap with other studies. (A) Venn diagram showing the number of overlapped genes that were significantly differentially expressed between the KS lesion and 3 other in vitro studies. Only genes with expression changes at FDR<5% in the same direction (lesion vs control or infected vs non-infected cells) were considered an overlap. (B) List of the 38 overlapped genes among the 4 studies. Lesion/control and infected/non-infected fold changes are shown. Red indicate up-regulation. Blue indicate down-regulation. (C) Genes overlapped with at least one study and are also involved in significantly enriched lipid and glucose functions.

Since most of the available KS or KSHV infection gene expression data has been derived from in vitro experiments in cell lines, we investigated the overlap of our in vivo lesion transcriptomes with these dataset derived from model systems (S4 Table). Gene expression data from 7 published studies were analyzed [41–47]. Only 3 of the 7 showed significant overlap with our data. Despite variability in the cell types employed in those 3 studies, our analysis indicated that 688 genes overlapped between our KS lesion dataset and these in vitro models (Fig 9A), with 38 overlapping genes having similar expression pattern among all 4 studies (Fig 9B). The KSHV infected TIME cells showed the highest similarity to lesions with 545 overlapped genes, followed by the LEC and HMVEC cells with 232 and 124 overlapped genes, respectively (Fig 9A). Some of these overlapped genes included, for example, a disintegrin and metalloprotease domain 19 (ADAM19) whose expression also correlated with KSHV RNA load in the lesion. In addition, the predicted upstream regulator erythroblast transformation-specific related gene (ERG) and one of the downstream affected genes C-X-C motif chemokine 11 (CXCL11) were also upregulated in in vitro models. Moreover, some of the overlapping genes among the studies also shared similar enriched functions, especially in glucose metabolism disorder and lipid functions (Fig 9C and S5 Table). However, overlapped genes represented only ~19% of the significantly differentially expressed genes in the lesion. The disparity in the gene expression profiles is likely due to the homogeneous nature of infected cell lines, whereas the lesion represented a much more complex, but realistic environment where multiple cell types co-exist and interact with direct and indirect effects of KSHV infection on cellular transcription.

## Discussion

This study is the first to profile the expression of KSHV and the cellular genes in the tumor microenvironment utilizing high-throughput RNA sequencing. Our data derived from lesion and contralateral control tissue biopsies of African epidemic KS patients showed a desultory pattern of KSHV gene expression in the tumor tissue between the patients. This is likely due to the multi-cellular lineage composition of the lesions, where diverse cell types in various stages of KSHV infection co-exist. However, we did find that the most dominantly expressed KSHV genes in all the lesions were involved in the establishment and maintenance of latency or in immune modulation. The high expression of viral immune modulation genes such as K2 (viral interleukin-6), K5 (modulator of immune recognition), K7 (viral inhibitor of apoptosis) and ORF75 (degradation of ND10 protein) in the lesions, could have severely hindered the immune system from eliminating these KSHV infected cells and contributed to the development of cancer. The upregulation of K2 in our data is consistent with a previous KS lesion gene expression study using real time-PCR [55]. In contrast to a prior report suggesting the potential oncogenic properties of the viral genes K1, ORF74 and vIRF1, we detected very few reads for these genes (ranged from 1 to 8 reads) in tumor tissues, suggesting either a very limited role of these genes in KS neoplastic growth or that their function is only essential at a temporally earlier stage in tumor development [56]. In addition, lytic gene expression was robust in the rapidly expanding cancer cells for two patients, p22 and p23 but restricted the other two patients. This observation is reminiscent of the limited sporadic lytic replication in various cell lines experimentally infected with KSHV, suggesting that sustained growth of KSHV transformed cells does not require robust lytic replication [57].

Interestingly, we found that patients with longer exposure to ART tended to have lower KSHV viral gene expression in their lesion and vice versa. For example, the patient p32 received ART for 60 months and had the least number of reads aligned to KSHV. The low viral gene expression also had a moderate inverse-correlation (*r* = −0.56) with higher lesion KSHV DNA copy number, as determined by real time-PCR. This suggests that prolonged ART treatment might suppress KSHV viral gene expression, but result in an increase of latent viral genome as shown by a higher KSHV DNA copy number in the lesions. A potential explanation is that the immune surveillance in these HIV-1 positive individuals improved after prolonged ART, effectively suppressing KSHV gene expression. However, a larger sample size will be needed to confirm these observed relationships.

Our study also revealed that expression of KSHV genes had a tremendous impact on the cellular gene expression profile. This is illustrated by the significant differences in gene expression between the lesion and control tissues that demonstrate KSHV mediated global transcriptional reprogramming in the lesion. Despite the disparity in KSHV gene expression, the cellular gene expression in the lesions was relatively similar between patients. By analyzing these common cellular gene changes with the IPA package, several upstream regulator genes were predicted to be altered in the lesions. These regulators, for example, upregulation of the transforming growth factor-beta 1 (TGFB1), could potentially impact a series of proteins and pathways involved in cell proliferation, differentiation and growth. Moreover, our analysis predicted the activation in lesions of expression of several CXCR3 chemokine ligands, CXCL-9, CXCL-10 and CXCL-11 that are known to play an important role in the recruitment of immune cells, and in particular, T cells. Interestingly, these 3 ligands were also reported to be upregulated in classic KS lesions, suggesting a potential universal phenomenon that is primarily driven by KSHV [58].

In agreement with the prediction of upregulation of CXCR3 chemokine ligands, CIBERSORT analysis implicated the lesion contained significant infiltration of various immune cells such as B cells, macrophages and natural killer cells in all patients. However, dendritic cells infiltration was predicted only in patients p32 and p83, whereas patients p22 and p23 had over 4-fold decrease than control tissues in apparent dendritic cells infiltration. It is worth noting that patients p32 and p83 had been on ART for an extended period of time, suggesting that ART might have stimulated some recovery of innate and adaptive anti-KSHV or anti-tumor immune response, but still was insufficient to suppress the KS development. The CIBERSORT also detected no significant infiltration of T cells in tumors, despite the apparent expression of the CXCR3 T cell chemo-attractants. This brings into question the functionality of the infiltrating immune cells. It is possible that these immune cells were able to migrate into the lesions but were incapable of activating their effector functions due to suppression by cytokines, anergy or checkpoint regulation [59]. Unfortunately, we were not able to confirm or refute these concepts using the current RNA-seq analyses alone. Transcriptomic, proteomic and functional assays on separate lineages of tumor-infiltrating lymphocytes with additional samples will need to be analyzed.

From the unique depth of the cellular transcriptomic data, we were also able to infer which biological functions in the lesions were most likely to have been affected by the alteration in cellular gene expression. Intriguingly, beyond a plethora of previously enumerated cancer-related functions, we discovered profound activation of glucose metabolism disorder, coupled with significant decreases in multiple lipid anabolic and catabolic pathways. The relationships between glucose and lipid metabolism in tumor is clearly complex, nevertheless, it is widely accepted that the Warburg effect, where multiple metabolic processes are altered in the tumor to sustain the rapid proliferation and expansion of cancerous cells, is common [60]. Our finding of an increased glucose metabolism disorder in KS lesions could be an indication of the Warburg effect and can be explained by the need for the cancer cells to replicate. Several studies have shown that KSHV is indeed capable to induce Warburg effect in cell-lines and resulted in up-regulation of metabolic pathways such as glycolysis [61–64]. Moreover, KSHV latency genes have been shown to affect glucose metabolism in vitro [65]. However, the decrease in lipid metabolism activities in these KS lesions is puzzling. Previous in vitro studies have suggested that KSHV requires and activates fatty acid synthesis for survival as latently infected endothelial cells. Additionally, multiple cellular genes (SCP2, PRDX5, ACSL3, MLYCD, AGPS, EHHADH, PEX19, PEX12, PEX5 and ABCD3) involved in lipid metabolism were up-regulated in these in vitro models [47, 66]. Increased lipid metabolism was also observed in other forms of cancer [67, 68]. Moreover, hypoxia conditions in the core of tumor should have stimulated lipid synthesis [69, 70]. Surprisingly, we observed a down-regulation of lipid metabolism genes in the context of KS lesions. Among the lipid metabolism genes identified in the previous studies [47, 66], we observed only marginal increased expression for ACSL3, AGPS and PEX12, the remaining genes were all down-regulated in our KS lesions [47]. Why lipid metabolism is not up-regulated, as suggested by in vitro models, in the KS lesions is unclear. Since KS lesion is a mixture of both normal and tumor cells, it is plausible that we are seeing an averaging effect with the KSHV-infected tumor cells undergoing up-regulated lipid biosynthesis, whereas the non-tumor cells were down-regulated. Interestingly, it had been reported that both ART and HIV-1 are capable of inducing lipid dysregulation, which is therefore a plausible cause for the decreased lipid metabolism observed in these epidemic KS patients [71–73]. It will be important to analyze the transcriptomes of lesions from HIV-1 negative endemic KS patients in order to differentiate the impacts due to ART, HIV-1 and KSHV alone on these metabolic changes.

Given the lack of suitable animal models for KSHV pathogenesis, in vitro cultures have been the only feasible mechanism to address the effects of KSHV on the infected cells. However, in vitro systems may not recapitulate the unique and complex microenvironment within the lesions. By comparing our lesion-based transcriptomic database with several in vitro studies, we showed that the TIME model reported recently is the most similar in its cellular gene expression profile to KS tumor biopsies. For example, the downregulation of peroxisome proliferator activated receptor gamma (PPARG) that regulates glucose metabolism and fatty acid storage was observed in both studies. Nevertheless, only 19% overlap between the lesion transcriptomes and in vitro derived expression repertoires was detected. More than 2901 genes were uniquely expressed in the lesions but not observed in the KSHV infected cell line models, suggesting that one needs to be cautious in extrapolating the gene expression data from cell lines to the human disease.

Finally, although the ipsilateral /contralateral control tissue collected from each KS patient served as an ideal intra-patient comparator for the lesion, the transcriptomes from these control tissues may not represent the gene expression profile of a KSHV negative patient. It is possible that the gene expression of these control tissues was already indirectly or directly altered by KSHV or by chronic immune responses. Skin tissue from KSHV negative patients will need to be tested to address this issue. Despite this shortfall, our data provide unique initial insights into the close relationship between the cellular and KSHV genes in tumorigenesis, as well as the profound impact of KSHV on numerous cellular regulatory and metabolic pathways that could potentially be targeted for future treatment of KS patients.

## Supporting information

S1 Table Primers for real time-PCR validation. (DOCX)

S2 Table Comparison of gene expression among the 4 lesion/control pairs. (XLSX)

S3 Table Genes involved in lipid-related functions and/or glucose metabolism disorder in lesions. (XLSX)

S4 Table In vitro studies compared with the current study. (DOCX)

S5 Table Comparison of gene expression and functions with other in vitro studies. (XLSX)

S1 Fig Top 30 most differentially expressed cellular genes in the lesions. (TIFF)
