# Supplementary material for: RNA-Seq of Kaposi’s sarcoma reveals alterations in glucose and lipid metabolism
Source: PLoS Pathog. 2018 Jan 19;14(1):e1006844. doi: 10.1371/journal.ppat.1006844 (PMC5792027; doi:10.1371/journal.ppat.1006844)
Supplement: S1 Table — (DOCX) [file ppat.1006844.s001.docx]

| **Host gene** | **Primer sequence** | **Tm (°C)** | **GC (%)** | **Amplicon Size (bp)** |
| --- | --- | --- | --- | --- |
| GAPDH | Forward - 5’ GAGTCCACTGGCGTCTTCAC 3’  Reverse - 5’ ATGACGAACATGGGGGCATC 3’ | 60.67  60.47 | 60  55 | 110 |
| TIE1 | Forward - 5’ GACAGTGGGTTCTGGGAGTG 3’  Reverse - 5’ CTCTGCTTGGTCAGGAGCC 3’ | 59.96  60.08 | 60  63.16 | 122 |
| CRYAB | Forward - 5’ GGAAAACATGAAGAGCGCCA 3’  Reverse - 5’ CCCCATCAGATGACAGGGATG 3’ | 59.12  59.93 | 50  57.14 | 121 |
| ADIPOQ | Forward - 5’ AGGGCATCCGGGCCATAAT 3’  Reverse - 5’ TCCGGTTTCACCGATGTCTC 3’ | 61.16  59.75 | 57.89  55 | 121 |
| ITGA9 | Forward - 5’ GGCTGTGTTTAAGTGCCGTG 3’  Reverse - 5’ ATCCACTCATCATCGCGGTC 3’ | 60.04  59.97 | 55  55 | 129 |
| ADAM19 | Forward - 5’ TTTCTCAGTTGGAGGCGCAA 3’  Reverse - 5’ ACTGGTACACAGAGCACATGG 3’ | 60.18  60 | 50  52.38 | 130 |
| PALD1 | Forward - 5’ GTGTTCGGCATGGGACAGC 3’  Reverse - 5’ ATCTGCACGCAGGAAAAGCA 3’ | 61.69  60.89 | 63.16  50 | 129 |
