# Supplementary material for: RNA-Seq of Kaposi’s sarcoma reveals alterations in glucose and lipid metabolism
Source: PLoS Pathog. 2018 Jan 19;14(1):e1006844. doi: 10.1371/journal.ppat.1006844 (PMC5792027; doi:10.1371/journal.ppat.1006844)
Supplement: S4 Table — (DOCX) [file ppat.1006844.s004.docx]

| **Study Set** | **Gene Set enrichment (GSE)** | **First and Last Authors** | **Title** | **Sequencing Type** | **Sub** | **Number of replicates** | **Cell** | **Virus** |
| --- | --- | --- | --- | --- | --- | --- | --- | --- |
| **1** | GSE1377 | Hong, Detmar | Lymphatic reprogramming of blood vascular endothelium by Kaposi sarcoma-associated herpesvirus | MA  Affymetrix U133A | GDS940 | 4 | HDMEC | JSC-1 |
| **2** | GSE6489 | Bull, Geraci | Human herpesvirus-8 infection of pulmonary microvascular endothelial cells | MA  Affy U133_Plus_2 | GDS3310 | 6 | HMVEC-L | BCBL-1 |
| **3** | GSE45590 | Chang, Ganem | A unique herpesviral transcriptional program in KSHV-infected lymphatic endothelial cells leads to mTORC1 activation and rapamycin sensitivity | MA  Agilent-029238, KSHV | GSE45590 | 2 | LEC | r219 |
|  |  |  |  |  |  | 2 | BEC | r219 |
| **4** | GSE66682 | Lee, Lee | The role of Kaposi’s sarcoma-associated herpesvirus infection in the proliferation of human bladder cancer cells | MA  Agilent-026652 | GSE66682 | 1 (2) | HT-1376 | BAC16 |
|  |  |  |  |  |  | 1 (2) | TCCSUP | BAC16 |
| **5** | GSE62344 | Purushothaman, Verma | Transcriptome Analysis of Kaposi’s sarcoma-associated herpesvirus during de novo primary infection of human B and endothelial-cells | RNA-seq | GSE62339 | 6 | PBMC | BAC36, BAC36d59 |
|  |  |  |  |  | GSE62340 | 5 | CD14+ | BCBL1 |
|  |  |  |  |  | GSE62341 | 5 | PBMC | BCBL1 |
|  |  |  |  |  | GSE62342 | 5 | TIVE | BCBL1 |
|  |  |  |  |  | GSE62343 | 3 | Virions | BCBL1, BAC36, BAC36d59 |
| **6** | GSE84237 | Sychev, Lagunoff | Integrated systems biology analysis of KSHV latent infection reveals viral induction and reliance on peroxisome mediated lipid metabolism | RNA-seq | GSE84237 | 6 | TIME | BCBL-1 |
| **7** | GSE56144 | Mercier, Ganem | Site-specific association with host and viral chromatin by Kaposi's sarcoma-associated herpesvirus LANA and its reversal during lytic reactivation | RNA-seq | GSE56144 | 4 | LEC | r219 |
