# Supplementary figures and images for: RNA-Seq of Kaposi’s sarcoma reveals alterations in glucose and lipid metabolism
Source: PLoS Pathog. 2018 Jan 19;14(1):e1006844. doi: 10.1371/journal.ppat.1006844 (PMC5792027; doi:10.1371/journal.ppat.1006844)

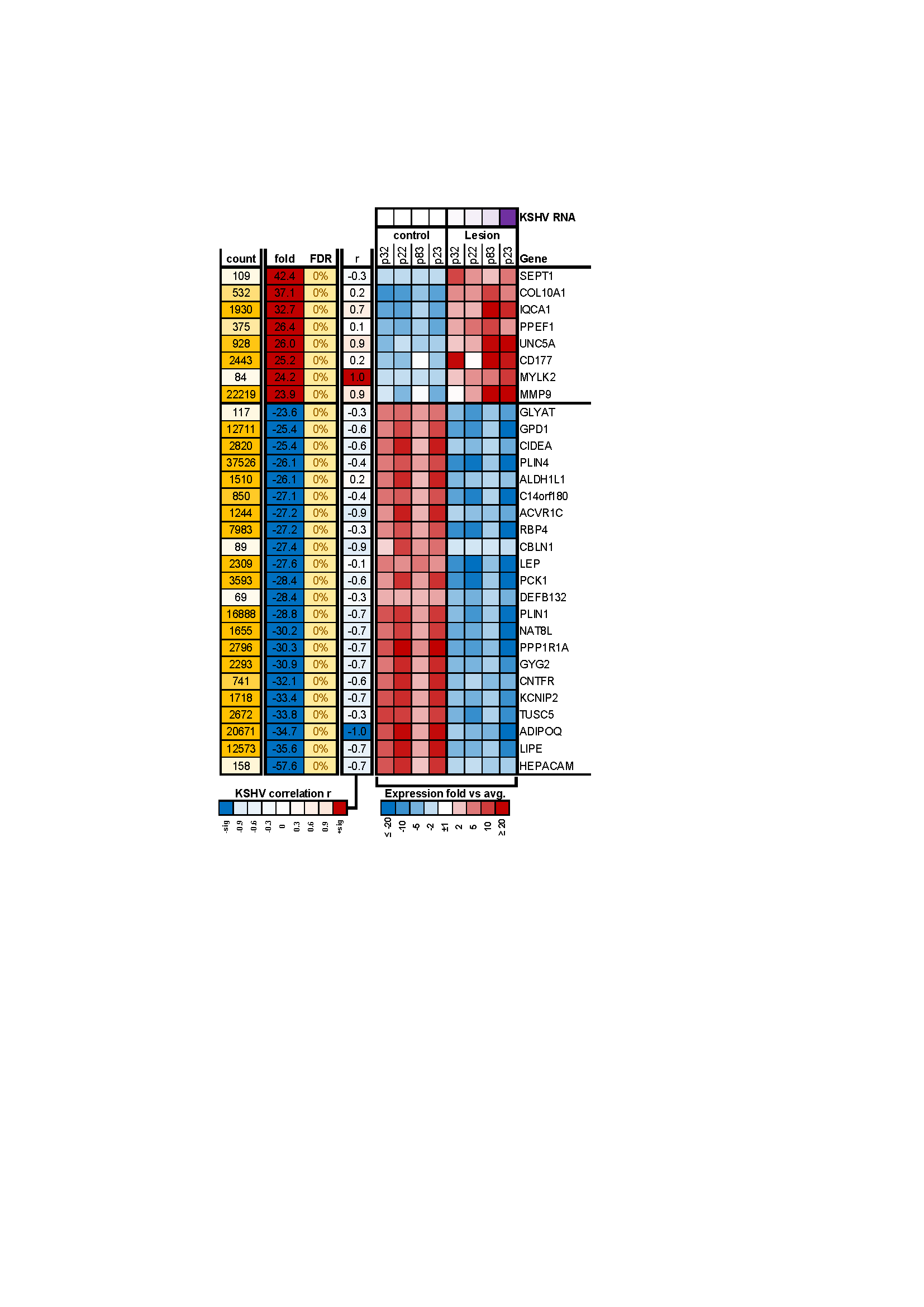

Supplement: S1 Fig — (TIFF) [file ppat.1006844.s006.tiff]
